# Morphological Spectrum of Glomerulonephritis in Medical Renal Biopsies: A Single-Center Study

**DOI:** 10.7759/cureus.22579

**Published:** 2022-02-24

**Authors:** Zubaria Rafique, Safana Sadaf, Saima Batool, Saira Javeed, Akhtar S Chughtai, Aribah Atiq

**Affiliations:** 1 Department of Pathology, Chughtai Institute of Pathology, Lahore, PAK

**Keywords:** nephrotic syndrome, lupus nephritis, fsgs, renal biopsy, chronic glomerulonephritis

## Abstract

Background

Glomerulonephritis is among the most common and serious non-communicable diseases in the world, and some of them are major causes of chronic kidney disease, which eventually leads to kidney failure. In developing countries, it is the most common cause of end-stage renal disease (ESRD). Chronic kidney disease affects 10-16% of the adult population in Asia, including 21.2% in Pakistan. This study aims to determine the frequency and spectrum of glomerulonephritis at our center.

Methodology

This is a cross-sectional analysis of 81 renal core biopsies obtained between August 2020 and August 2021. The histopathological reports, demographic data, and relevant laboratory investigations, such as blood urea and creatinine levels, were collected. All cases were blindly reviewed by two pathologists with a special interest in medical renal pathology. Data were analyzed using SPSS version 22 (IBM Corp., Armonk, NY, USA).

Results

The majority of the patients (46.9%) were between the ages of 21 and 40 years. There was a slight male predominance, with 44 (54.3%) of the 81 participants being male. Hematuria was reported in 20 (24.7%) patients, with mean serum urea and creatinine levels of 75 mg/dL and 2.6 mg/dL, respectively. Nephrotic syndrome was the most common indication for a renal biopsy, accounting for 54 (67.7%) of the 81 cases. Chronic glomerulonephritis is classified into two categories, namely, primary and secondary glomerulonephritis. Focal segmental glomerulosclerosis (FSGS), the leading cause of primary glomerulonephritis, was found in 25 (30.9%) 81 patients. Furthermore, lupus nephritis (9.9%) and advanced glomerulonephritis (9.9%) were found in equal proportions among secondary glomerulonephritis.

Conclusions

According to our study, nephrotic syndrome was the most common indication for medical renal biopsy, with FSGS being the most common primary glomerulonephritis. Lupus nephritis and advanced glomerulonephritis were the most common secondary glomerulonephritis diagnoses.

## Introduction

Among non-communicable diseases, renal diseases are considered to be one of the most common and serious maladies globally [1]. The incidence of kidney disease continues to rise and is among the major causes of soaring morbidity [2].

Renal ailments are usually signs of chronic kidney disease, which ultimately leads to kidney failure. One such disease is chronic glomerulonephritis, which is the most common cause of end-stage renal disease (ESRD) in developing countries [3]. Due to diverse environmental, ethnic, social, and rural-urban disparities, the prevalence of chronic kidney disease varies greatly around the world. In Pakistan, the epidemiology of chronic kidney disease and its risk factors are poorly understood, and there has been little research on this topic [4]. Chronic kidney disease presently affects 10-16% of the adult population in Asia [5]. Its prevalence among the Pakistani population is 21.2% [6].

A renal biopsy is a primary mode of establishing and confirming the diagnosis in the majority of chronic kidney diseases. It is an accurate and gold-standard tool for diagnosing glomerular diseases and determining the prognosis of patients. Furthermore, immunofluorescence (IF) studies provide additional information for histopathological analysis [7]. Currently, almost 99.9% of biopsies are diagnostic and result in an accurate diagnosis [8].

Pathologically, different types of renal diseases can vary considerably with factors such as patients’ ethnicity, age group, sex, and geographical location/origin [9].

In many developed countries, renal biopsy registries have been established and are functioning at full scale to document the varying trends in the disease range. However, developing countries like Pakistan lack such registries and face a woeful scarcity of data on renal diseases [10]. The objective of this study is to assess the frequency and spectrum of glomerulonephritis in our population at the Chughtai Institute of Pathology, Lahore.

## Materials and methods

This is a cross-sectional study of 81 renal core biopsies received at the Chughtai Institute of Pathology between August 2020 and August 2021. All cases were extracted from archives of Chughtai lab using Nexus software. Renal core biopsies of patients of all age groups and both genders with renal insufficiency (decreased glomerular filtration rate and deranged renal functions) were included along with demographic details, laboratory investigations, such as 24-hour urinary protein, serum urea, and creatinine levels, and histopathology reports. Inadequate or scanty biopsies with no glomeruli, biopsies with suboptimal fixation, and transplant biopsies were excluded from the study. Each biopsy was evaluated using the histologic adequacy criterion of more than or equal to 10 glomeruli.

In this study, 10% neutral buffered formalin was used for the fixation of renal core biopsy for light microscopy evaluation, and normal saline was used for IF studies. The core biopsy fixed in 10% buffered formalin was processed in an automated tissue processor (Peloris, Leica, Germany) and then cut at 3 µ thickness to prepare standard glass slides. Subsequently, the slides were stained with hematoxylin and eosin (H&E), Jones’ methenamine silver stain (JMS), periodic acid-Schiff (PAS), and Masson’s trichrome stains (Biognost, Merck, USA).

The core biopsy fixed in normal saline was processed as frozen tissue in a cryostat machine at -20° for IF assessment. Following the embedding of tissue in optimal cutting temperature compound, glass slide preparation included cutting the tissue at a thickness of 3 µ, fixing it in alcohol, air drying it for 10 minutes, and treating it with phosphate buffer saline (PBS) at a pH of 7.2 for 30 minutes. Fluorescein isothiocyanate-conjugated polyclonal rabbit antisera against human immunoglobulin (Ig)G, IgM, IgA, C3, C1q (Dako, Glostrup, Denmark) were used to stain slides for IF microscopy. Positive and negative controls were also performed. After five minutes in PBS wash buffer, the slides were mounted using a fluorescent mounting medium. The slides were then examined through the green filter of the IF microscope, and direct IF findings were interpreted based on the intensity, pattern, and distribution of immune deposits. Electron microscopy was not performed due to the lack of facility at our center. Two pathologists with a special interest in medical renal pathology blindly reviewed all cases.

SPSS version 22 (IBM Corp., Armonk, NY, USA) was used to analyze the data. The numerical variables in descriptive analyses are reported as means and standard deviations, whereas frequencies or categorical variables are expressed as percentages.

## Results

The mean age in our study population was 29.2 (±16.07) years. The age range was 3-73 years, with the most common age group being 21-40 years, accounting for 46.9% of the total with 38 patients (Table 1). There were 25 (30.9%) patients in the age group 1-20 years, and 14 (17.3%) patients in the age group 41-60 years. There were four patients in the elderly group of 61-80 years (4.9%).

**Table 1:** Demographics and clinical parameters for kidney biopsies.

Demographic and clinical parameters	Number (%)
Gender	
Male	44 (54.3%)
Female	37 (45.7%)
Age range	
1–20 years	25 (30.9%)
21–40 years	38 (46.9%)
Renal parameters	
Serum creatinine, mean mg/dL	2.6
Serum urea, mean mg/dL	75
Renal biopsy indication	
Massive proteinuria (>3.5 g/24 hours)	54 (66.7%)
Sub-nephrotic-range proteinuria (<3.5 g/24 hours)	27 (33.3%)
Hematuria	20 (24.7%)

Of the 81 cases, males outnumbered females (males: 54.3%, females: 45.7%). In this study, the mean serum urea and creatinine levels were 75 (±54.6) mg/dL and 2.6 (±2.5) mg/dL, respectively. Hematuria was reported in 20 (24.7%) patients. The most common indication for renal biopsy was nephrotic syndrome (66.7%), followed by sub-nephrotic-range proteinuria (33.3%) (Table 1). In a total of 81 cases, the average number of glomeruli was 14.4 (±8.3).

Interstitial fibrosis and tubular atrophy (IFTA) is a histological hallmark of chronic kidney disease progression. All cases were scored for IFTA, with 41 (50.7%) in the mild IFTA category. Moderate IFTA was found in 29 (35.8%) 81 cases, with severe IFTA found in eight (9.8%). Three (3.7%) cases out of 81 had minimal or no IFTA (Table 2).

**Table 2:** IFTA scoring. IFTA: interstitial fibrosis and tubular atrophy

IFTA	Total number	Percentage (%)
Minimal, <10%	03	3.7%
Mild, <25%	41	50.7%
Moderate, 25–50%	29	35.8%
Severe, >50%	08	9.8%

Among the primary glomerulonephritis, focal segmental glomerulosclerosis (FSGS) (30.9%) was the most commonly observed diagnosis, followed by membranous glomerulonephritis (23.5%) and membranoproliferative glomerulonephritis (6.2%) (Table 3). According to the Columbia classification, FSGS is classified into five morphological variants, namely, collapsing, tip, perihilar, cellular, and not otherwise specified (NOS). In our study population, there were 25 cases of FSGS, with eight NOS, seven perihilar, six cellular, and four tip variants. None of the cases contained the collapsing variant. In secondary causes of glomerulonephritis, lupus nephritis and advanced glomerulosclerosis (9.9%) were the most common (Table 3).

**Table 3:** Frequency of histological diagnosis of primary and secondary glomerulonephritis. FSGS: focal segmental glomerulosclerosis; MEM: membranous glomerulonephritis; MPGN: membranoproliferative glomerulonephritis; IgAN: immunoglobulin A nephropathy; MCD: minimal change disease; GN: glomerulonephritis

	Frequency (%)
Primary glomerulonephritis	
FSGS	25 (30.9%)
MEM	19 (23.5%)
MPGN	5 (6.2%)
IgAN	2 (2.5%)
MCD	1 (1.2%)
Secondary glomerulonephritis	
Lupus nephritis	8 (9.9%)
Advanced GN	8 (9.9%)
Amyloidosis	5 (6.2%)
Post-infectious GN	5 (6.2%)
Diabetic nephropathy	3 (3.7%)

## Discussion

Glomerulonephritis is an inflammation of the glomeruli, which are tiny blood-filtering structures in the kidney. It is a more common cause of chronic kidney disease in developing countries and is caused by many conditions, which include immune system abnormalities, genetic disorders, infections, and drugs. Early diagnosis can help in the effective cure and control of this disease to a large extent. The spectrum of glomerulonephritis has changed worldwide in the last few decades. Demographic, social, and economic status account for the changing spectrum of glomerulonephritis [11].

Nephrotic syndrome (massive proteinuria >3.5 g/24 hours, hypoalbuminemia, and edema), nephritic syndrome (hematuria, proteinuria, and hypertension), rapidly progressive glomerulonephritis (marked hematuria), asymptomatic urinary abnormalities, acute kidney injury, and chronic kidney disease are clinical indications for biopsy [12].

According to the World Health Organization classification, glomerulonephritis is divided into two major categories: (1) Primary glomerulonephritis (caused by factors intrinsic to the kidney, which are often idiopathic and without accompanying conditions) includes minimal change disease, IgA nephropathy, membranous, membranoproliferative, and FSGS. (2) Secondary glomerulonephritis (associated with a factor or underlying systemic diseases such as infections, drugs, and metabolic diseases) includes lupus nephritis, diabetes, amyloidosis, post-infectious, and advanced glomerulonephritis [9].

In the current study, 81 renal biopsies were analyzed from August 2020 to August 2021. A male predominance was noted in our study, with 44 (54.3%) male patients and 37 (45.7%) female patients. This was consistent with other studies conducted globally, including Saudi Arabia, China, and India [9,13,14]. In a Chinese survey, male predominance was observed to be 56% [14].

In our study, the population was divided into four age groups. Among them, glomerulonephritis is more common in the younger population in the age group of 21 to 40 years. The number of patients in the group was 38, accounting for 46.9% of the entire data. Another study conducted in China by Liangmei et al. also reported similar results, with most patients between the ages of 15 and 49 years, accounting for 52.3% of the total study population [14]. The second group of patients, aged 1 to 20 years, had 25 patients, accounting for 30.9% of the total, followed by 14 (17.3%) patients between the ages of 41 and 60 years. The minimum number of cases (4.9%) were seen in the elderly group aged 61 to 80 years.

Nephrotic syndrome was the most common reason for a renal biopsy, with proteinuria greater than 3.5 g/24 hours in 54 of the 81 patients, accounting for 66.7% of the cases. The most common symptoms of nephrotic syndrome were generalized edema and frothy urine. This was followed by sub-nephrotic-range proteinuria of less than 3.5 g/24 hours. Our results are in concordance with other studies conducted globally. According to a study conducted in Taiwan by Hsien et al., the most prevalent clinical presentation was nephrotic syndrome, which was reported by 36.1% of patients [13,15,16].

In our study population, 20 of the 81 patients had hematuria, accounting for 24.7% of the cases. The mean serum creatinine and urea levels were 2.6 (±2.5) mg/dL and 75 (±54.6) mg/dL, respectively, which is consistent with a comparable study conducted in India by Josephine et al., which found increased serum urea and creatinine levels in 47.7% and 66.6% of subjects, respectively [13].

Primary glomerulonephritis

IgA nephropathy is the most common primary glomerulonephritis worldwide [9,17,18]. On reviewing the literature of the spectrum of primary glomerular diseases, IgA nephropathy is more common in developed countries such as Saudi Arabia, Kuwait, Poland, Singapore, and the Czech Republic [19-21]. However, IgA nephropathy ranks fourth in our analysis, accounting for only 2.5% of primary glomerulonephritis. Our findings are in line with those of Umesha et al., who found IgA nephropathy in 13.4% of cases in research conducted in India [22].

**Figure 1:**
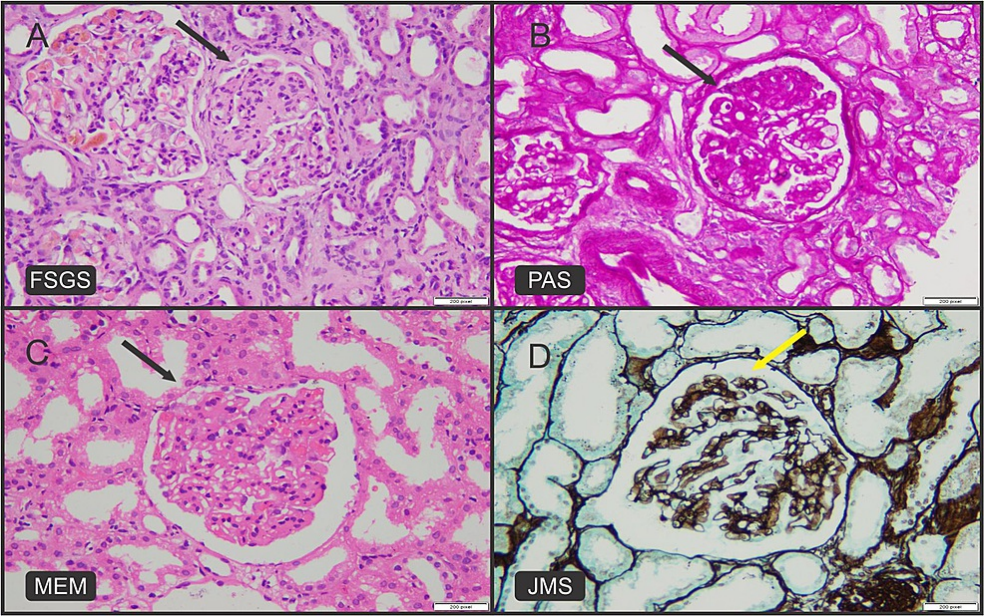
(A) H&E image shows segmentally sclerosed glomerulus in FSGS (black arrow). (B) PAS highlights segmentally sclerosed glomerulus in FSGS (black arrow). (C) H&E image shows thickened glomerular capillary loops in membranous glomerulonephritis (black arrow). (D) JMS highlights spikes in membranous glomerulonephritis (yellow arrow). H&E: hematoxylin and eosin; FSGS: focal segmental glomerulosclerosis; PAS: periodic acid–Schiff; JMS: Jones’ methenamine silver stain; MEM: membranous glomerulonephritis

In contrast, FSGS was the most frequent diagnosis and the leading cause of nephrotic syndrome in our study population, accounting for 25 (30.9%) of the total 81 patients (Figures 1A, 1B). Similar rising trends were observed in studies conducted in India, which revealed that FSGS (12%) is the most frequent primary glomerulonephritis [13,22,23]. It indicates that FSGS is more frequent in developing countries. According to two studies conducted in Pakistan and Jordan, membranous glomerulonephritis is the most prevalent primary glomerulonephritis, followed by FSGS [10,11]. In our study population, however, membranous glomerulonephritis was the second most common cause of primary glomerulonephritis (Figures 1C, 1D).

In our study cohort, we found an increased number of FSGS (30.9%) and membranous glomerulonephritis (23.5%), highlighting the need for further research to find the prevalence of associated variables such as hepatitis B and HIV infections in at-risk populations [24].

Secondary glomerulonephritis

**Figure 2:**
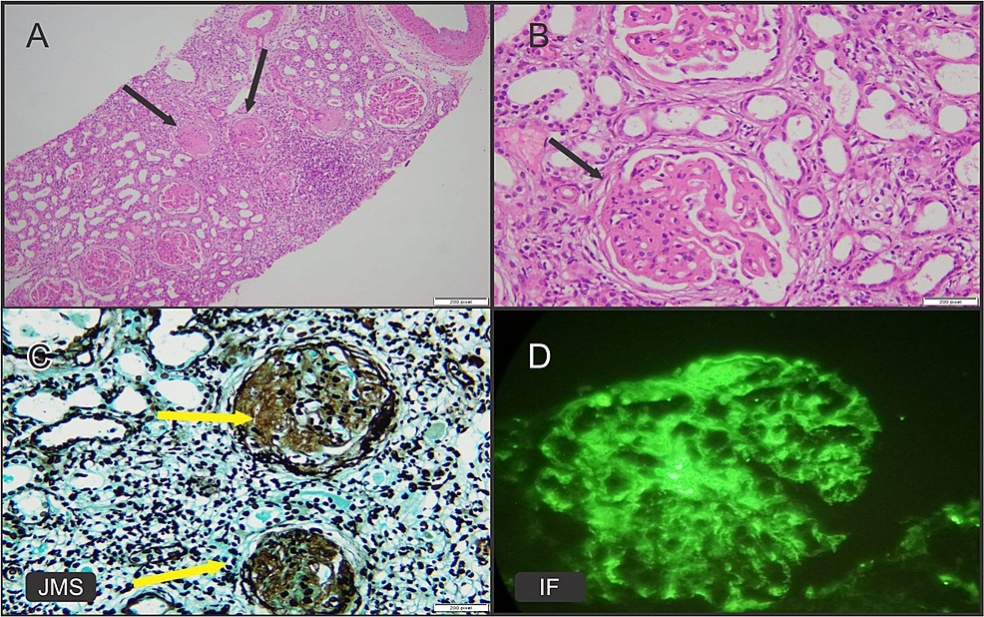
Lupus nephritis, class V. (A) H&E image shows globally thickened glomeruli (black arrows) and leukocytic infiltration. (B) H&E high-power view of glomerulus showing thickened capillary loops and membranes (black arrow). (C) JMS highlights thickened glomerular capillary loops (yellow arrows). (D) Immunofluorescence shows granular IgG immune complex deposits in mesangium and capillary loops. H&E: hematoxylin and eosin; JMS: Jones’ methenamine silver stain; IF: immunofluorescence; Ig: immunoglobulin

In our study, lupus nephritis and advanced glomerulonephritis were the predominant patterns among secondary glomerulonephritis, representing 9.9% of the cases (Figures 2A-2D). In the present series, lupus nephritis with evidence of female predominance was reported in eight (9.9%) cases, which are compatible with several other studies [11,14,15] (Figures 2A-2D). According to a study conducted in the Czech Republic by Dita et al., 23.2% of secondary glomerulonephritis patients have lupus nephritis [21]. Lupus nephritis is the frequent cause of secondary glomerulonephritis and remains universal globally [18]. Another study conducted in Pakistan by Muhammad et al. found that advanced glomerulonephritis (24%), after lupus nephritis (30.3%), was the second most prevalent cause of secondary glomerulonephritis [10]. The fact that advanced glomerulonephritis cases account for the same proportion of lupus nephritis cases in our population demonstrates that patients are diagnosed late due to a lack of awareness of disease progression and consequences.

Limitations

There are certain limitations to our research, such as the lack of electron microscopy and light chain kappa and lambda antibodies for IF at our center, precluding further assessment. Furthermore, because it was a single-center study, the results may not reflect the true frequency of glomerulonephritis in other regions.

## Conclusions

The main indication of renal biopsy was the nephrotic syndrome, while FSGS was reported as the most common primary glomerulonephritis, followed by membranous glomerulonephritis. Lupus nephritis and advanced glomerulonephritis were the most frequent diagnoses among secondary glomerulonephritis. Our study will be helpful in providing epidemiological data regarding changes in the spectrum of primary and secondary glomerulonephritis in our population. By having a comprehensive understanding of the evolving pattern of glomerulonephritis, clinicians can focus on future clinical trials addressing the underlying etiologies, pathophysiology, and most relevant therapeutics.
